# Supplementary material for: Synthetic accessibility and stability rules of NASICONs
Source: Nat Commun. 2021 Oct 1;12:5752. doi: 10.1038/s41467-021-26006-3 (PMC8486869; doi:10.1038/s41467-021-26006-3)
Supplement: Supplementary file 1 — Supplementary Information [file 41467_2021_26006_MOESM1_ESM.pdf]

Synthetic accessibility and stability rules of NASICONs

Supplementary information

Bin Ouyang[1,2,†], Jingyang Wang[1,2,†], Tanjin He[1,2], Christopher J. Bartel[1,2], Haoyan Huo[1,2], Yan Wang[3], Valentina Lacivita[3], Haegyeom Kim[1] and Gerbrand Ceder[1,2]*

[1] Materials Sciences Division, Lawrence Berkeley National Laboratory, Berkeley, CA 94720, USA

[2] Department of Materials Science and Engineering, University of California, Berkeley, CA 94720, USA

[3] Advanced Material Lab, Samsung Research America, 10 Wilson Rd., Cambridge, MA 02138, USA

† These authors contributed equally to this work

* corresponding author:

Prof. Gerbrand Ceder (Email: gceder@berkeley.edu)

Supplementary note 1. Evaluation of ideal entropy of mixing (S_{ideal})

The partially occupied sites of NASICON are considered for ideal entropy calculations. For a NASICON with the chemical formula $Na_xM_yM'_{2-y}(AO_4)_z(BO_4)_{3-z}$, the ideal entropy is calculated as below with the unit of eV/atom:

$$S_{ideal} = -k_b \frac{1}{x+17}\left(\frac{1}{4}\left(\frac{x}{4}\ln\left(\frac{x}{4}\right) + (1-\frac{x}{4})\ln\left(1-\frac{x}{4}\right)\right) + \frac{1}{2}\left(\frac{y}{2}\ln\left(\frac{y}{2}\right) + (1-\frac{y}{2})\ln\left(1-\frac{y}{2}\right)\right) + \frac{1}{3}\left(\frac{z}{3}\ln\left(\frac{z}{3}\right) + (1-\frac{z}{3})\ln\left(1-\frac{z}{3}\right)\right)\right)$$

Supplementary note 2. E_{hull} and calculated competing phases of six synthesis attempts of NASICONs

Table S1. Six newly predicted and experimentally synthesized Na-rich silicon phosphate NASICON compounds

#	Formula	E_{hull} (meV/atom)	Competing Phases
1	$Na_3HfZr(SiO_4)_2(PO_4)$	1.9	$Na_4P_2O_7$ + $HfSiO_4$ + $Na_3Zr_2(SiO_4)_2(PO_4)$ + $Na_2ZrSi_2O_7$ + HfO_2
2	$Na_3Hf_{1.5}Mg_{0.5}(SiO_4)(PO_4)_2$	8.0	$NaMgPO_4$ + $NaHf_2(PO_4)_3$ + $Na_4P_2O_7$ + $HfSiO_4$ + HfO_2
3	$Na_3HfSn(SiO_4)_2(PO_4)$	12.6	$HfSiO_4$ + $Na_4P_2O_7$ + $Na_2Si_2O_5$ + SnO_2
4	$Na_3HfSc(SiO_4)(PO_4)_2$	15.0	$HfSiO_4$ + $Na_4P_2O_7$ + $Na_3Sc_2(PO_4)_3$ + $NaSc(SiO_3)_2$ + HfO_2
5	$Na_3Ca_{0.5}Hf_{1.5}(SiO_4)(PO_4)_2$	17.1	$HfSiO_4$ + $NaCaPO_4$ + $Na_4P_2O_7$ + $NaHf_2(PO_4)_3$ + HfO_2
6	$Na_3Mg_{0.5}Zr_{1.5}(SiO_4)(PO_4)_2$	17.4	$Na_4Mg_3P_4O_{15}$ + $Na_4P_2O_7$ + $NaZr_2(PO_4)_3$ + $ZrSiO_4$ + ZrO_2

Supplementary note 3. Comparisons of E_{hull} values for Monoclinic and Rhombohedral NASICONs.

#	Formula	E_{hull}^{Robh} (meV/atom)	E_{hull}^{Mono} (meV/atom)	$\|E\_diff\|$ (meV/atom)
1	$Na_3Hf_2(SiO_4)_2(PO_4)$	-5.2	-8.5	3.3
2	$Na_3Sc_2(PO_4)_3$	10.1	6.4	3.7
3	$Na_3MgZr(PO_4)_3$	7.3	6.8	0.5
4	$Na_3Al_2(PO_4)_3$	7.6	7.8	0.2
5	$Na_3Zr_2(SiO_4)_2(PO_4)$	12.2	11.7	0.5
6	$Na_3Y_2(PO_4)_3$	13.3	12.7	0.4
7	$Na_3Mg_{0.5}Zr_{1.5}(SiO_4)(PO_4)_2$	17.4	21.2	3.8
8	$Na_3MgTi(PO_4)_3$	19.5	18.4	1.1
9	$Na_3In_2(PO_4)_3$	21.6	18.7	2.9
10	$Na_3HfZr(SiO_4)_2(PO_4)$	4.1	1.9	2.2
11	$Na_3HfSc(SiO_4)(PO_4)_2$	15.0	15.2	0.2
12	$Na_3Hf_{1.5}Mg_{0.5}(SiO_4)(PO_4)_2$	8.0	11.8	3.8
13	$Na_3Mg_{0.5}Zr_{1.5}(SiO_4)(PO_4)_2$	17.4	21.2	3.8
14	$Na_3Ca_{0.5}Hf_{1.5}(SiO_4)(PO_4)_2$	17.1	17.9	0.8
15	$Na_3HfSn(SiO_4)_2(PO_4)$	16.1	12.6	3.5

Supplementary note 4. Experimentally reported NASICON conductivity

Table S2. Summary of Na^+ conductivity plotted in Figure 4a. *: conductivity was calculated according to the Arrhenius equation. †: conductivity was measured at 327 °C.

Composition	σ_{RT} (S/cm)	$\sigma_{300°C}$ (S/cm)	Reference
NaZr(PO4)3	4.50E-06	4.89E-05	1[1]
Na1.4Zr2P2.6Si0.4O12	8.49E-06*	5.36E-04	2[2]
Na1.8Zr2P2.2Si0.8O12	1.85E-05*	2.93E-03	2
Na2.2Zr2P1.8Si1.2O12	3.47E-04*	2.63E-02	2
Na2.6Zr2P1.4Si1.6O12	2.50E-04*	4.76E-02	2
Na2.8Zr2P1.2Si1.8O12	2.86E-03*	1.25E-01	2
Na3Zr2PSi2O12	1.82E-03*	2.00E-01	2
Na3.2Zr2P0.8Si2.2O12	3.83E-03*	1.67E-01	2
Na3.4Zr2P0.6Si2.4O12	1.76E-03*	1.11E-01	2
Na3.6Zr2P0.4Si2.6O12	8.18E-04*	3.57E-02	2
Na3.8Zr2P0.2Si2.8O12	4.10E-04*	1.79E-02	2
Na4Zr2(SiO4)3	8.87E-09	1.10E-03	1
NaTi2P3O12		2.30E-05†	3[3]
Na1.4Ti1.6In0.4P3O12		4.86E-04†	3
NaSn2P3O12		6.40E-07†	3
Na1.4Sn1.65In0.4P3O12		6.13E-04†	3
NaHf2P3O12		4.90E-05†	3
Na1.4Hf1.6In0.4P3O12		3.59E-04†	3
Na1.4Zr1.6In0.4P3O12		2.41E-03†	3
Na3.1Sc0.1Zr1.9Si2PO12	1.32E-03		4[4]
Na3.2Sc0.2Zr1.8Si2PO12	1.90E-03		4
Na3.3Sc0.3Zr1.7Si2PO12	2.80E-03		4
Na3.4Sc0.4Zr1.8Si2PO12	4.00E-03		4
Na3.5Sc0.5Zr1.5Si2PO12	3.80E-03		4
Na3.6Sc0.6Zr1.8Si2PO12	1.75E-03		4
Na3.05La0.05Zr1.95Si2PO12	9.60E-04	1.42E-01	5[5]
Na3.10La0.1Zr1.9Si2PO12	1.30E-03	1.32E-01	5
Na3.15La0.15Zr1.85Si2PO12	1.50E-03	1.27E-01	5
Na3.2La0.2Zr1.8Si2PO12	1.80E-03	1.58E-01	5
Na3.25La0.25Zr1.75Si2PO12	3.20E-03	1.59E-01	5
Na3.3La0.3Zr1.7Si2PO12	3.40E-03	1.52E-01	5
Na3.35La0.35Zr1.65Si2PO12	2.00E-03	1.13E-01	5
Na3.4La0.4Zr1.6Si2PO12	1.30E-03	9.00E-02	5
Na2.85In1.85Zr0.15(PO4)3	3.30E-06		6[6]
Na1.8In0.8Zr1.2(PO4)3	5.30E-07		6
Na2InZr(PO4)3	1.00E-06		6
Na2.5In1.5Zr0.5(PO4)3	2.36E-06		6
Na2.85Zr2Si1.85P1.15O12		2.10E-01	7[7]
Na3.3Zr2Si2.3P0.7O12		2.00E-01	7
Na1.45Zr1.55Yb0.45(AsO4)3	2.00E-06		8[8]
Na1.35Zr1.65Yb0.35(AsO4)3	1.41E-06		8

Na1.25Zr1.75Yb0.25(AsO4)3	1.00E-06		8
Na1.15Zr1.85Yb0.15(AsO4)3	7.10E-07		8
NaZr2(AsO4)3	4.00E-07		8
Na2.8Zr1.7Nd0.3Si1.5P1.5O12	1.90E-04	5.30E-02	9[9]
Na3Sc2(PO4)3	1.05E-06	1.39E-02	10[10]
Na3MnZr(PO4)3	1.80E-06	2.00E-03	11[11]
Na3MgZr(PO4)3	1.00E-06	1.20E-03	11
Na3Zr2Si2P0.8As0.2O12	2.08E-03*	2.29E-01*	12[12]
Na3Zr2Si2P0.6As0.4O12	1.36E-03*	1.79E-01*	12
Na3Zr2Si1.6Ge0.4PO12	2.39E-03*	1.51E-01*	12
Na3Zr2Si1.2Ge0.8PO12	2.07E-03*	1.57E-01*	12
Na3Zr2Si1.6Ge0.4P0.8As0.2O12	1.58E-03*	2.09E-01*	12
Na3Zr2Si1.2Ge0.8P0.6As0.4O12	1.50E-03*	1.37E-01*	12
Na3Zr1.6Ti0.4Si2PO12	6.82E-04*	1.30E-01*	12
Na3Zr1.2Th0.8Si2PO12	2.75E-03*	1.44E-01*	12
Na3Zr1.2Hf0.8Si2PO12	4.23E-03*	1.85E-01*	12
Na3Hf2Si2PO12	5.48E-03*	1.99E-01*	12
Na2ZrYb(PO4)3	1.20E-05		13[13]
Na2.5Zr0.5Cr1.5(PO4)3	2.40E-05		13
Na2YZr(PO4)3	1.60E-07		14[14]
Na2.25Y1.25Zr0.75(PO4)3	1.00E-06		14
Na2.5Y1.5Zr0.5(PO4)3	2.50E-06		14
Na2.75Y1.75Zr0.25(PO4)3	6.31E-06		14
Na3Y2(PO4)3	5.60E-06		14
Na3Zr1.98Mg0.02Si2PO12		1.60E-01	15[15]
Na3Zr1.98V0.02Si2PO12		1.40E-01	15
Na3Zr1.98Nb0.02Si2PO12		1.30E-01	15
Na3Zr1.98Ta0.02Si2PO12		1.10E-01	15
Na2.5Zr1.8Sc0.2Si1.3P1.7O12	2.00E-04	4.50E-02	16[16]
Na1.5Ga0.5Zr1.5(PO4)3		4.50E-04	17[17]
Na1.5Cr0.5Zr1.5(PO4)3		1.77E-03	17
Na1.5Al0.5Zr1.5(PO4)3		5.00E-05	17
Na1.5Sc0.5Zr1.5(PO4)3		3.55E-03	17
Na1.5In0.5Zr1.5(PO4)3		3.47E-03	17
Na1.5Yb0.5Zr1.5(PO4)3		1.78E-03	17
Na1.5Y0.5Zr1.5(PO4)3		2.51E-03	17
Na3Zr1.9Nb0.08Si2PO12	2.06E-04	9.94E-02	18[18]
Na3Zr1.7Nb0.24Si2PO12	2.26E-04	1.06E-01	18
Na3Zr1.5Nb0.40Si2PO12	1.20E-04	3.93E-02	18
Na3Zr1.3Nb0.56Si2PO12	3.31E-05	1.21E-02	18
Na3Zr1Nb0.8Si2PO12	3.31E-05	1.74E-03	18
Na1.2Zr1.9Mg0.1P3O12	6.90E-06	4.70E-05	19[19]
Na1.6Zr1.7Mg0.3P3O12	8.90E-06	7.50E-05	19
Na2.0Zr1.5Mg0.5P3O12	9.60E-06	1.30E-04	19
Na2.4Zr1.3Mg0.7P3O12	1.40E-05	1.60E-04	19
NaSn2(PO4)3	1.50E-05		20[20]
Na1.6Al0.2Hf1.8Si0.4P2.6O12	4.33E-05		21[21]
Na2.2Al0.4Hf1.6Si0.8P2.2O12	1.06E-04		21
Na2.8Al0.6Hf1.4Si1.2P1.8O12	5.85E-04		21

Na3.4Al0.8Hf1.2Si1.6P2.4O12	2.11E-04		21
Na4AlHfSi2PO12	1.92E-04		21
Na1.5Nb0.3Zr1.5(PO4)3	1.70E-09	1.23E-04	22[22]
Na3Zr1.88Y0.12Si2PO12	2.70E-03		23[23]
Na1.4Al0.4Ti0.6(PO4)3	1.00E-07		24[24]
Na1.4Al0.4Sn0.6(PO4)3	1.00E-08		24
Na1.4Al0.4Ge0.6(PO4)3	6.30E-10		24
Na1.3Al0.3Ge1.7P3O12		6.77E-05	25[25]
Na1.4Al0.4Ge1.6P3O12		7.11E-05	25
Na1.6Al0.6Ge1.4P3O12		1.58E-04	25
Na1.7Al0.7Ge1.4P3O12		7.10E-05	25
Na1.8Al0.8Ge1.2P3O12		3.80E-04	25
Na3.1Zr1.95Mg0.05Si2PO12	3.50E-03		26[26]
Na3.1Zr1.95Ca0.05Si2PO12	2.10E-03		26
Na3.1Zr1.95Sr0.05Si2PO12	1.80E-03		26
Na3.1Zr1.95Ba0.05Si2PO12	1.30E-03		26
Na3.2Zr1.8Ca0.2Si2PO12	1.67E-03		27[27]
Na3.38Zr1.80Al0.26Si2.06P0.88O12	4.39E-04		28[28]
Na3.13Zr1.94Fe0.21Si2.12P0.85O12	7.53E-04		28
Na3.24Zr1.91Y0.09Si1.93P1.02O12	3.52E-04		28
Na3.47Zr1.93Co0.18Si1.97P0.91O12	1.55E-04		28
Na3.59Zr1.92Ni0.21Si1.93P0.91O12	6.18E-04		28
Na3.43Zr1.83Zn0.22Si1.93P1.02O12	8.05E-04		28
Na3.256Mg0.128Zr1.872Si2.2P0.8O12	2.70E-03		29[29]
Na3.25Zr1.9375Ni0.0625Si2.2P0.8O12	2.10E-03		29
NaFe2(PO4)(SO4)2		3.50E-08	30[30]
Na2.4Hf2(SiO4)1.4(PO4)1.6	7.30E-04	1.27E-01	1, 31[31]
Na2.5Hf2(SiO4)1.5(PO4)1.5	1.87E-04	1.34E-02	1, 31
Na2.6Hf2(SiO4)1.6(PO4)1.4	5.90E-04	1.42E-01	1, 31
Na2.8Hf2(SiO4)1.8(PO4)1.2	6.90E-04	1.57E-01	1, 31
Na3Hf2(SiO4)2(PO4)	1.10E-03	1.51E-01	1, 31
Na3.2Hf2(SiO4)2.2(PO4)0.8	2.30E-03	2.24E-01	1, 31
Na3.4Hf2(SiO4)2.4(PO4)0.6	1.40E-03	7.68E-02	1, 31
Na3.6Hf2(SiO4)2.6(PO4)0.4	1.20E-03	5.76E-02	1, 31
Na3.8Hf2(SiO4)2.8(PO4)0.2	3.20E-04	4.31E-02	1, 31
Na3.5Hf0.2Ti1.8(SiO4)1.5(PO4)1.5	3.36E-04	3.52E-02	1
Na3.5Hf0.6Ti1.4(SiO4)1.5(PO4)1.5	2.81E-04	3.61E-02	1
Na3.5HfTi(SiO4)1.5(PO4)1.5	1.35E-04	3.47E-02	1
Na3.5Hf1.4Ti0.6(SiO4)1.5(PO4)1.5	8.70E-05	1.19E-02	1
Na3Hf0.2Ti1.8(SiO4)2(PO4)	2.32E-04	1.33E-02	1
Na3Hf0.4Ti1.6(SiO4)2(PO4)	9.60E-05	8.40E-03	1
Na3Hf0.6Ti1.4(SiO4)2(PO4)	2.84E-04	1.48E-02	1
Na3Hf0.8Ti1.2(SiO4)2(PO4)	1.78E-04	7.00E-03	1
Na3HfTi(SiO4)2(PO4)	1.31E-04	6.50E-03	1
Na3Ti0.1Zr1.9(SiO4)2(PO4)	1.10E-04	6.90E-02	1
Na3Ti0.2Zr1.8(SiO4)2(PO4)	8.75E-05	5.50E-02	1
Na3Ti0.3Zr1.7(SiO4)2(PO4)	9.87E-05	6.20E-02	1
NaGe2(PO4)3	1.10E-12	2.56E-06	1
NaTi2(PO4)3	4.43E-10	2.98E-05	1

NaHf2(PO4)3	8.77E-10	1.03E-04	1
NaGe0.5Ti1.5(PO4)3	5.91E-13	8.70E-07	1
NaGeTi(PO4)3	8.50E-12	2.01E-06	1
NaGe1.5Ti0.5(PO4)3	3.13E-11	8.90E-06	1
NaSn0.5Ti1.5(PO4)3	1.77E-11	1.29E-06	1
NaSnTi(PO4)3	6.86E-11	3.64E-06	1
NaSn1.5Ti0.5(PO4)3	5.14E-10	6.11E-06	1
NaSn0.5Zr1.5(PO4)3	4.23E-11	2.70E-06	1
NaSnZr(PO4)3	2.47E-10	1.58E-05	1
NaSn1.5Zr0.5(PO4)3	7.91E-10	2.40E-05	1
NaNbZr(PO4)3	2.49E-08	3.10E-04	1
NaNbTi(PO4)3	1.59E-06	1.00E-03	1
NaMoZr(PO4)3	2.06E-09	3.10E-05	1
NaMoTi(PO4)3	3.07E-07	2.80E-04	1
Na3.08Mg0.04Zr1.96(SiO4)2(PO4)	3.70E-04	1.60E-01	1
Na3.2Mg0.1Zr1.9(SiO4)2(PO4)	2.08E-04	6.20E-02	1
Na3.6Mg0.3Zr1.7(SiO4)2(PO4)	9.02E-05	3.90E-02	1
Na3.08Zn0.04Zr1.96(SiO4)2(PO4)	2.19E-05	3.50E-02	1
Na3.2Zn0.1Zr1.9(SiO4)2(PO4)	8.44E-05	4.40E-02	1
Na3.4Zn0.2Zr1.4(SiO4)2(PO4)	5.05E-04	4.90E-02	1
Na3.6Zn0.3Zr1.7(SiO4)2(PO4)	1.75E-05	2.80E-02	1
Na3.28Mg0.04Zr1.96(SiO4)2.2(PO4)0.8	8.06E-04	2.40E-01	1
Na3.36Mg0.08Zr1.92(SiO4)2.2(PO4)0.8	4.64E-04	9.50E-02	1
Na3.52Mg0.16Zr1.84(SiO4)2.2(PO4)0.8	1.71E-04	2.00E-02	1
Na3.36Zn0.08Zr1.92(SiO4)2.2(PO4)0.8	1.10E-03	2.10E-01	1
Na3.36Cu0.08Zr1.92(SiO4)2.2(PO4)0.8	5.31E-04	2.09E-01	1
Na3.36Co0.08Zr1.92(SiO4)2.2(PO4)0.8	1.03E-03	2.40E-01	1
Na3.232Co0.016Zr1.984(SiO4)2.2(PO4)0.8	1.41E-03	2.40E-01	1
Na3.52Co0.16Zr1.84(SiO4)2.2(PO4)0.8	5.88E-04	1.00E-01	1
Na1.2In0.2Zr1.8(PO4)3	2.08E-07	1.46E-03	1
Na1.4In0.4Zr1.6(PO4)3	9.76E-07	3.25E-03	1
Na1.5Al0.5Zr1.5(PO4)3	5.70E-06	6.70E-04	1
Na1.5Cr0.5Zr1.5(PO4)3	1.00E-05	5.50E-04	1
Na1.5Ga0.5Zr1.5(PO4)3	3.40E-06	3.40E-04	1
Na1.5In0.5Zr1.5(PO4)3	2.90E-05	1.90E-03	1
Na1.5Sc0.5Zr1.5(PO4)3	5.80E-05	2.30E-03	1
Na1.5Y0.5Zr1.5(PO4)3	5.60E-05	1.40E-03	1
Na1.5Yb0.5Zr1.5(PO4)3	3.00E-05	1.20E-03	1
Na2AlZr(PO4)3	1.20E-06	2.30E-04	1
Na2CrZr(PO4)3	2.50E-05	8.50E-04	1
Na2.2In1.2Zr0.8(PO4)3	2.63E-06	4.99E-03	1
Na2.3Yb1.3Zr0.7(PO4)3	6.03E-06	5.50E-03	1
Na2.5Cr1.5Zr0.5(PO4)3	1.80E-04	7.20E-03	1
Na2.5In1.5Zr0.5(PO4)3	1.00E-04	6.40E-03	1
Na2.5Sc1.5Zr0.5(PO4)3	5.60E-04	1.20E-02	1
Na2.5Y1.5Zr0.5(PO4)3	4.60E-05	2.50E-03	1
Na2.5Yb1.5Zr0.5(PO4)3	1.90E-04	2.40E-02	1
Na2.5Er1.5Zr0.5(PO4)3	1.24E-09	1.00E-04	1
Na2.5Dy1.5Zr0.5(PO4)3	1.65E-08	6.30E-04	1

Na2.6In1.6Zr0.4(PO4)3	2.81E-06	1.13E-02	1
Na2.8In1.8Zr0.2(PO4)3	2.38E-06	5.45E-03	1
Na2.85In1.85Zr0.15(PO4)3	5.25E-07	3.10E-03	1
Na2.95Cr1.95Zr0.05(PO4)3	4.22E-06	9.80E-03	1
Na3Cr2(PO4)3	1.70E-07	7.00E-03	1
Na3Fe2(PO4)3	1.20E-07	9.00E-03	1
Na3In2(PO4)3	1.76E-07	9.00E-04	1
Na3Sc2(PO4)3	2.27E-05	5.68E-02	1
Na1.4Al0.4Ge1.6(PO4)3	7.28E-10	6.98E-05	1
Na1.4Al0.4Sn1.6(PO4)3	1.41E-08	4.42E-04	1
Na1.4Al0.4Ti1.6(PO4)3	5.60E-08	1.43E-03	1
Na1.6Al0.6Ti1.4(PO4)3	1.10E-07	2.57E-03	1
Na1.8Al0.8Ti1.2(PO4)3	1.20E-07	2.74E-03	1
Na1.9Al0.9Ti1.1(PO4)3	1.30E-07	1.82E-03	1
Na1.4In0.4Ti1.6(PO4)3	1.86E-08	3.03E-04	1
Na1.4In0.4Sn1.6(PO4)3	2.72E-08	4.04E-04	1
Na1.4In0.4Hf1.6(PO4)3	1.86E-07	2.67E-04	1
Na2.04Y0.04Zr1.96(SiO4)2(PO4)	2.08E-04	6.20E-02	1
Na2.1Y0.1Zr1.9(SiO4)2(PO4)	1.98E-03	1.10E-01	1
Na2.3Y0.3Zr1.7(SiO4)2(PO4)	9.76E-05	2.00E-02	1
Na2.5Sc0.2Zr1.8(SiO4)1.3(PO4)1.7	3.19E-04	4.50E-02	1
Na3Sc1.5Zr0.5(SiO4)0.5(PO4)2.5	1.17E-04	2.90E-02	1
Na3ScZr(SiO4)2(PO4)	1.82E-04	3.10E-02	1
Na3Sc0.8Zr1.2(SiO4)1.2(PO4)1.8	1.42E-04	2.90E-02	1
Na3Sc0.5Zr1.5(SiO4)1.5(PO4)1.5	1.41E-04	1.07E-01	1
Na3Sc0.2Zr1.8(SiO4)1.8(PO4)1.2	1.90E-04	1.20E-01	1
Na3.3Sc0.3Zr1.7(SiO4)2(PO4)	4.14E-04	1.79E-01	1
Na3.5Sc0.5Zr1.5(SiO4)2(PO4)	4.88E-04	1.00E-01	1
Na2.7Sc0.2Zr1.8(SiO4)1.5(PO4)1.5	8.87E-05	8.10E-02	1
Na2.96Nb0.04Zr1.96(SiO4)2(PO4)	4.95E-03	1.30E-01	1
Na2.9Nb0.1Zr1.9(SiO4)2(PO4)	1.87E-03	4.90E-02	1
Na2.96Ta0.04Zr1.96(SiO4)2(PO4)	9.40E-04	1.10E-01	1
Na2.9Ta0.1Zr1.9(SiO4)2(PO4)	5.30E-04	6.20E-02	1
Na2.96V0.04Zr1.96(SiO4)2(PO4)	1.20E-03	1.40E-01	1
Na2.9V0.1Zr1.9(SiO4)2(PO4)	4.78E-04	9.80E-02	1
Na3Sc0.25Zr1.75(SiO4)1.75(PO4)1.25	1.58E-05	1.18E-02	32[32]
Na1.5Al0.5Ge1.5(PO4)3	3.20E-07		33[33]
Na3.4Sc2(SiO4)0.4(PO4)2.6	6.90E-04		34[34]
Na3Zr1.9Yb0.1Si2PO12	3.70E-04		35[35]
Na3Zr1.9Gd0.1Si2PO12	5.50E-04		35
Na3Zr1.9Ce0.1Si2PO12	8.20E-04		35
Na1.1Al0.05Y0.05Zr1.9(PO4)3	1.10E-06		36[36]
Na1.2Al0.1Y0.1Zr1.8(PO4)3	5.00E-06		36
Na1.3Al0.15Y0.15Zr1.7(PO4)3	6.00E-06		36
Na1.4Al0.30Y0.30Zr1.4(PO4)3	6.20E-06		36
Na3.1Al0.05Y0.05Zr1.9(SiO4)2(PO4)	1.60E-03		36
Na3.2Al0.1Y0.1Zr1.8(SiO4)2(PO4)	1.30E-03		36
Na3.4Al0.2Y0.2Zr1.6(SiO4)2(PO4)	1.40E-03		36
Na3.6Al0.3Y0.3Zr1.6(SiO4)2(PO4)	4.60E-04		36

Supplementary note 5. Calculated COHP and kernel density for analyzing bond compatibility and site miscibility.

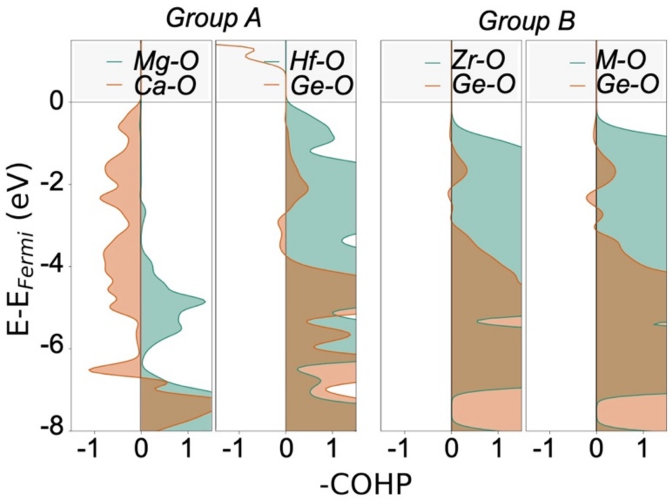

Figure S1: Calculated -COHP normalized by number of metal-O bonds for four comparison groups, i.e., A/A1, A/A2, B/B1 and B/B2 from left to right

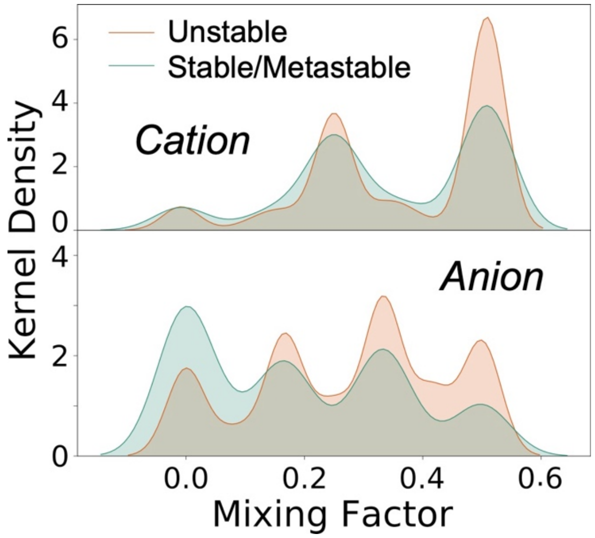

Figure S2: Kernel distribution of NASICONs as a function of cation/anion mixing factor. The mixing factor is defined as $(y/2, 1-y/2)$ for cation mixing and $min(z/3, 1-z/3)$ for anion mixing.

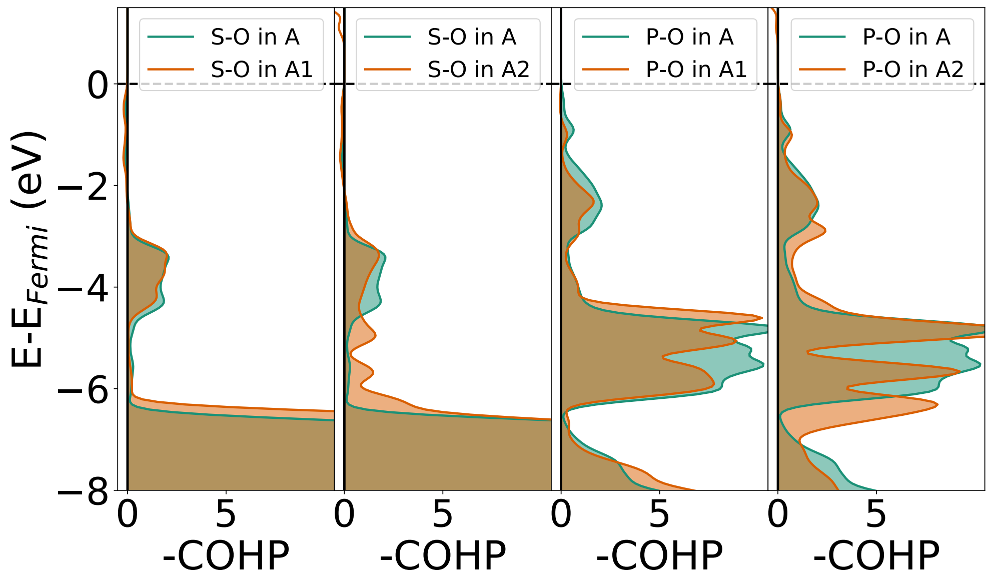

Figure S3: Calculated -COHP normalized by number of S-O/P-O bonds for A/A1 and A/A2 comparisons.

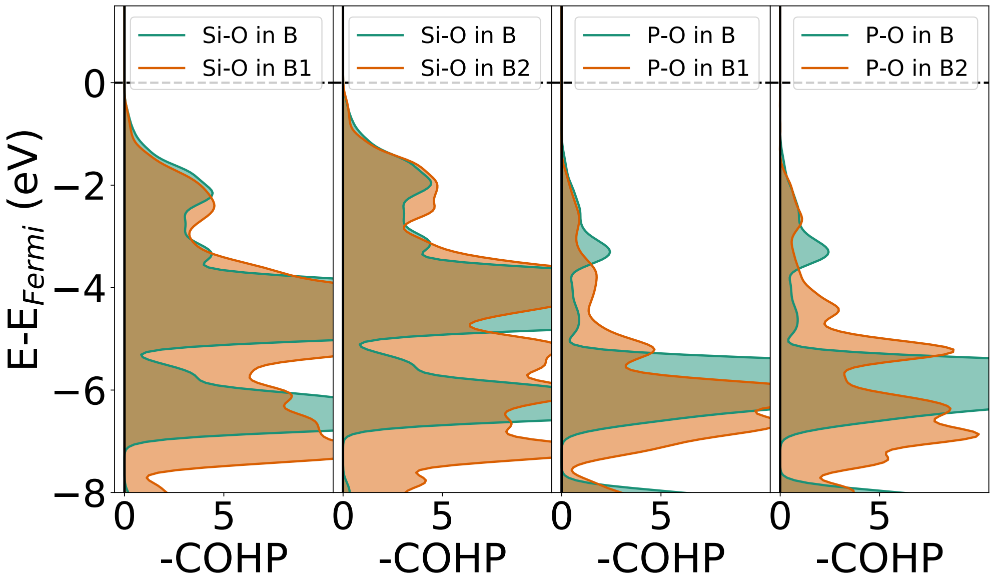

Figure S4: Calculated -COHP normalized by number of Si-O/P-O bonds for A/A1 and A/A2 comparisons.

Table S3: Calculated ICOHP normalized by number of ions for A/A1/A2 and B/B1/B2 comparisons.

ICOHP	M-O	A-O
A ($NaHfMg(SO_4)_2(PO_4)$)	1.22	7.34
A1 ($NaHfCa(SO_4)_2(PO_4)$)	0.92	7.38
A2 ($NaGeMg(SO_4)_2(PO_4)$)	0.74	6.59
B ($Na_3HfZr(SiO_4)_2(PO_4)$)	4.47	10.63
B1 ($Na_3HfGe(SiO_4)_2(PO_4)$)	3.18	10.71
B2 ($Na_3Ge_2(SiO_4)_2(PO_4)$)	1.38	10.91

Supplementary note 6. More details of the machine learning process

The properties related to stability of NASICON would be electronegativity (X), ionic radius (R), ionic charge states (Q), Na content (N_{Na}), formation energy of competing phases (E_{Form}), Ewald summation energy (E_{Ewald}). These basic properties can be obtained from different sites, and with different mathematical form. For Pauling electronegativity (X), Shannon ionic radius (R) and ionic charge states (Q), both metal sites and anion sites are investigated, leading to $X_{M/A}$, $R_{M/A}$ and $Q_{M/A}$, respectively. In addition, the average across all sites, standard deviation, maximum deviation were considered, which are noted as X_M^{Avg}, X_M^{Std}, X_M^{Std}, X_A^{Avg}, X_A^{Std}, X_A^{Std}, R_M^{Avg}, R_M^{Std}, R_M^{Std}, R_A^{Avg}, R_A^{Std}, R_A^{Std}, Q_M^{Avg}, Q_M^{Std}, Q_M^{Std}, Q_A^{Avg}, Q_A^{Std}, Q_A^{Std}. For ionic radius and electronegativity, three additional features were considered, i.e., R_M^{Avg}/R_{Na}, $X_M^{Avg} - X_{Na}$ and $X_M^{Avg} - X_A$. For the anion site, the property of center ion i.e., Si^{4+}, P^{5+} and S^{6+}, was also considered Therefore, using the aforementioned $E_{Form,}$ N_{Na} and E_{Ewald}, a total of 24 basic features are constructed. To enlarge the physically meaningful searching space for mathematical representations, all ionic radii are normalized with the radius of Na^+, while all charge states are normalized by the charge of an electron.

It is worth mentioning that the real E_{Ewald} energy is related to not only Na contents and average cation/anion charge states, but also cation/anion ordering and lattice parameter. Here we did not want to involve the impacts of cation/anion ordering and lattice parameter, as those properties cannot be estimated without first principal calculations. The E_{Ewald}^2 we calculated was on base of a structural model with the lattice parameter of $Na_3Zr_2(SiO_4)_2(PO_4)$ and electrostatic ground state ionic ordering. Similarly, the E_{Ewald} value is normalized by the Ewald energy of $Na_3Zr_2(SiO_4)_2(PO_4)$. Therefore, this parameter can be interpreted as a dimensionaless physical term that measures the electrostatic ground-state electrostatic interaction as a function of Na

content and average cation/anion charge states. It can then be used as a descriptor that couples the influenced Na contents and cation/anion charge states. The structural model and an example python script for calculating E_{Ewald} value is appended as "NASICONPrim.cif" and "CalculateEWaldEnergy.ipynb" respectively.

Since the SISSO code only allows mathematical operation with complexity of 3 or less, we have tried complexity from 1 to 3 and found that a complexity of 2 gives the best trade-off between validation accuracy and simplicity, as increasing the complexity from 2 to 3 increase the validation accuracy with less than 1%.

It should be noted that selecting the final model from screening 2 million possible models may raise the concern about overfitting. According to PAC theory[37-39], to reduce the risk of overfitting (measured by generalization error ε), the amount of training data m should follow $m \propto O\left(\frac{1}{\varepsilon^2} * \mathrm{VC}(H)\right)$, where $VC(H)$ refers to the Vapnik–Chervonenkis (VC) dimension of our SIS+MLR classification model. Even though we have considered 2 million candidate models to find the best solution, we still have quite limited complexity of our model selection as (a) we use only 24 features to generate each descriptor, (b) we combine features with only 17 basic mathematical operators, and (c) the final model contains only two fit coefficients. By doing this, the maximal complexity of each descriptor is fixed, and so is $\mathrm{VC}(H)$[37-39]. Hence, the risk of overfitting is largely reduced.

Supplementary note 7. Model performance metrics of SVC classification. The model generated from the 3rd is chosen as the final model:

Table S4. Metrics of testing set in 5-fold cross validation.

	1st fold	2nd fold	3rd fold	4th fold	5th fold
Accuracy	83.0%	80.7%	81.3%	84.5%	80.9%
Recall (LS/GS)	87.6%	82.8%	81.3%	92.2%	81.3%
Recall (US)	82.1%	80.2%	81.3%	83.0%	80.9%
Recall (Avg)	84.8%	81.5%	81.3%	87.6%	81.1%
F1 (LS/GS)	63.1%	58.6%	58.9%	66.3%	58.4%
F1 (US)	89.0%	87.4%	87.9%	90.0%	87.6%
F1 (Avg)	76.0%	73.0%	73.4%	78.1%	73.0%

Table S5. Metrics of training set in 5-fold cross validation.

	1st fold	2nd fold	3rd fold	4th fold	5th fold
Accuracy	82.0%	82.7%	82.7%	81.5%	82.4%
Recall (LS/GS)	84.4%	86.0%	85.6%	83.6%	86.5%
Recall (US)	81.5%	82.1%	82.2%	81.1%	81.6%
Recall (Avg)	82.9%	84.0%	83.9%	82.3%	84.1%
F1 (LS/GS)	60.7%	62.2%	62.1%	59.9%	61.9%
F1 (US)	88.3%	88.8%	88.8%	88.0%	88.6%
F1 (Avg)	74.5%	75.5%	75.5%	73.9%	75.2%

Supplementary note 8. Predicted probability for 32 experimentally synthesized NAISCONs

The calculated probabilities of the 32 experimentally synthesized NASICONs being GS/LS according to Platt scaling are indicated Table S3. The model used for generating the probabilities are the final mode shown in Fig. 5.

Table S6. Calculated Platt scaling probability for 32 experimentally synthesized NASICONs.

Composition	$P_{accessible}$	Classified	Composition	$P_{accessible}$	Classified
$Na_3Zr_2(SiO_4)_2(PO_4)$	0.428	No	$NaZnIn(SO_4)_3$	0.778	Yes
$Na_4Zr_2(SiO_4)_3$	0.496	No	$NaMgIn(SO_4)_3$	0.772	Yes
$NaTi_2(PO_4)_3$	0.888	Yes	$Na_3HfTi(SiO_4)_2(PO_4)$	0.322	No
$NaSn_2(PO_4)_3$	0.888	Yes	$NaGe_2(PO_4)_3$	0.888	Yes
$NaHf_2(PO_4)_3$	0.888	Yes	$NaTiGe(PO_4)_3$	0.760	Yes
$Na_3MgZr(PO_4)_3$	0.622	Yes	$NaTiSn(PO_4)_3$	0.748	Yes
$Na_2Zr_2(SiO_4)(PO_4)_2$	0.588	Yes	$NaZrSn(PO_4)_3$	0.855	Yes
$Na_3ZrTi(SiO_4)_2(PO_4)$	0.310	No	$Na_2ZrAl(PO_4)_3$	0.493	No
$Na_3Ti_2(SiO_4)_2(PO_4)$	0.428	No	$Na_3In_2(PO_4)_3$	0.622	Yes
$Na_3Sc_2(PO_4)_3$	0.622	Yes	$NaZr_2(PO_4)_3$	0.888	Yes
$Na_3ZrZn(PO_4)_3$	0.596	Yes	$Na_3Al_2(PO_4)_3$	0.622	Yes
$Na_3Hf_2(SiO_4)_2(PO_4)$	0.428	No	$Na_3HfZr(SiO_4)_2(PO_4)$	0.420	No
$Na_3Y_2(PO_4)_3$	0.622	Yes	$Na_3Hf_{1.5}Mg_{0.5}(SiO_4)(PO_4)_2$	0.421	No
$Na_4HfAl(SiO_4)_2(PO_4)$	0.185	No	$Na_3HfSc(SiO_4)(PO_4)_2$	0.397	No
$NaAlZn(SO_4)_3$	0.387	No	$Na_3Ca_{0.5}Hf_{1.5}(SiO_4)(PO_4)_2$	0.274	No
$NaMgAl(SO_4)_3$	0.583	Yes	$Na_3Mg_{0.5}Zr_{1.5}(SiO_4)(PO_4)_2$	0.428	No

Supplementary note 9. Distribution of P_{stable} of US NASICONs and GS/LS NASICONs across the SVC classification boundary.

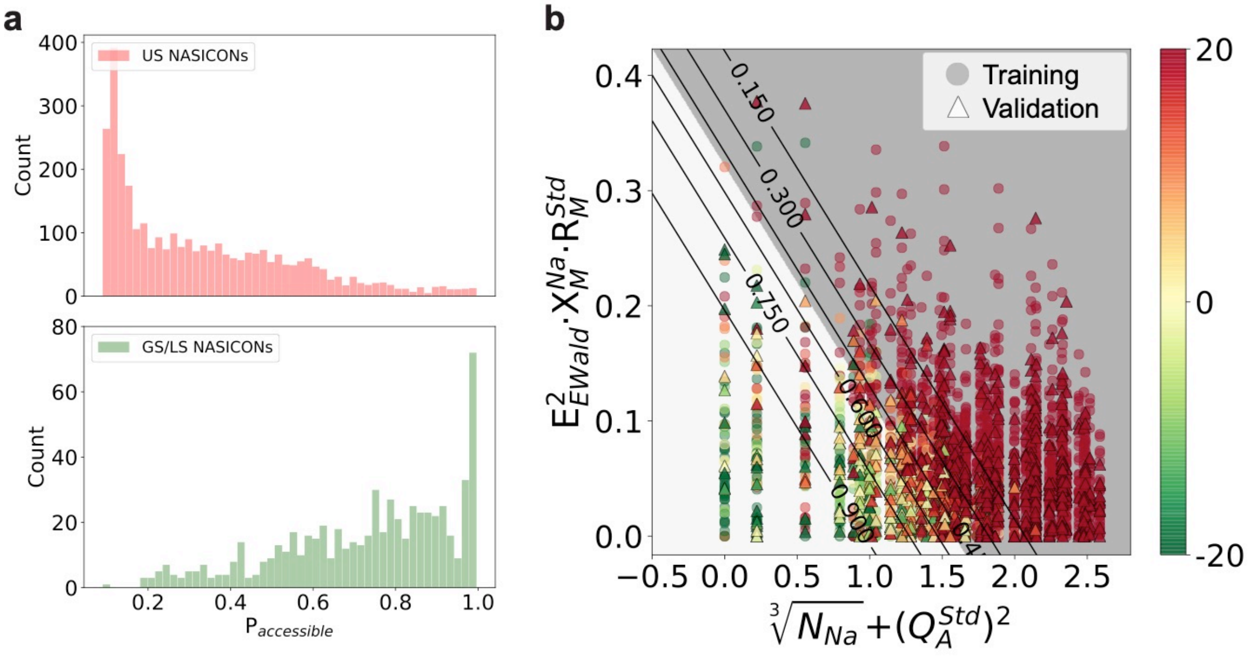

Figure S5. a. The histogram of P_{stable} for GS/LS NASICONs and US NASICONs as classified by the SVC classification boundary; **b.** The contour lines of P_{stable} near the SVC classification boundary.

Supplementary note 10. Predicted probability map for Na Poor/Na Rich NASICONs and Ca/Ge/Hf/Zr NASICONs

The predicted probability distributions of Na Poor/Na Rich NASICONs and Ca/Ge/Hf/Zr NASICONs are demonstrated in Figure S6:

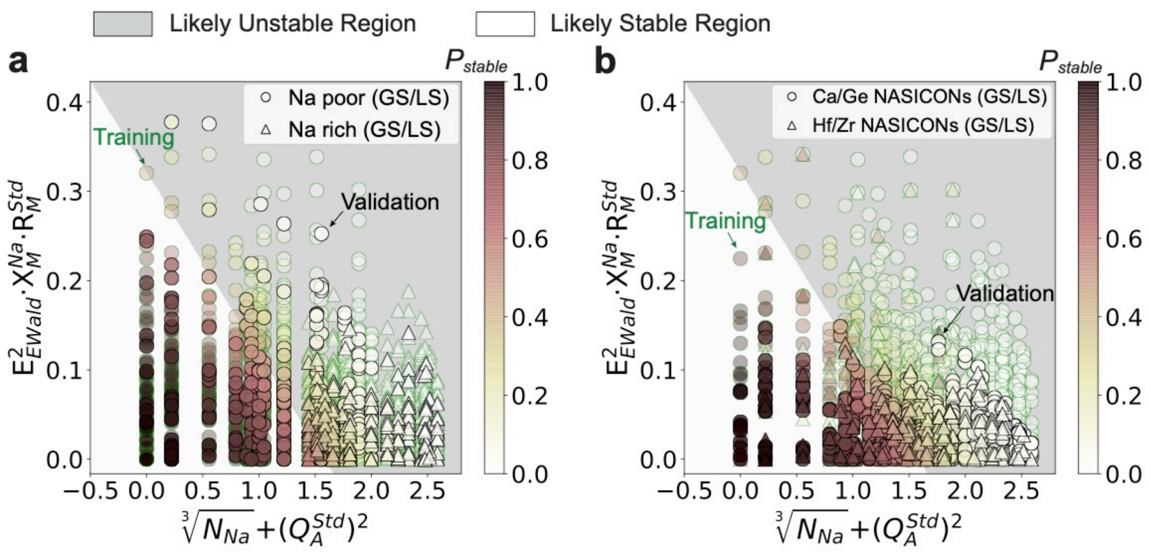

Figure S6: a. The Platt scaling probability to be accessible ($P_{accessible}$) distribution of Na rich and Na poor NASICONs; **b.** The Platt scaling accessible probability ($P_{accessible}$) distribution of Ca/Ge/Zr/Hf contained NASICONs.

Supplementary note 11. Comparisons between -ICOHP of M-O bonds in B, B1, B2 and the -ICOHP of the M-O bonds in the competing phases of B1 and B2

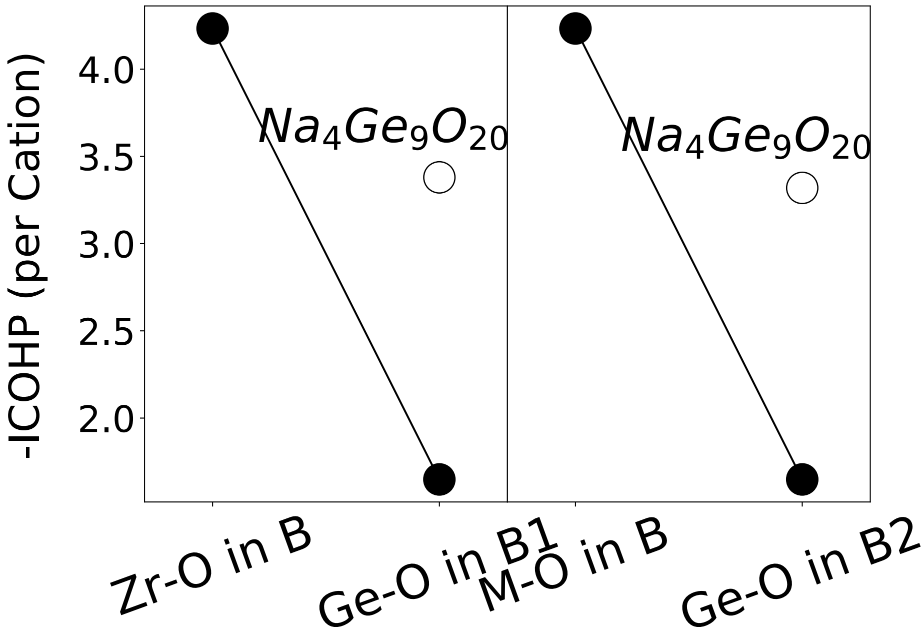

Figure S7. The calculated -ICOHP for different M-O bonds in NASICONs composition B, B1 and B2. As B1 and B2 are predicted to be unlikely synthesizable (E_{hull} = 56.0 meV/atom and 90.3 meV/atom respectively), the calculated -ICOHP per cation of the M-O bonds in competing phase is also plotted.

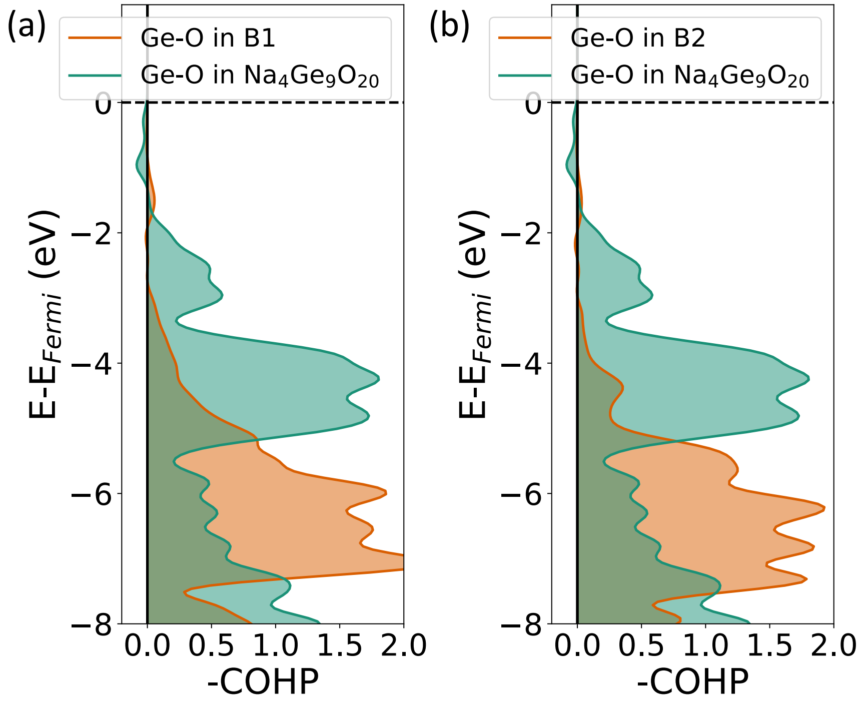

Figure S8. The calculated -COHP comparisons (normalized by M-O bond number) for (**a**) Ge-O in B1 and $Na_4Ge_9O_{20}$; (**b**) Ge-O in B2 and $Na_4Ge_9O_{20}$.

Supplementary note 12. Comparisons between -ICOHP in A, A1, and the Ca contained competing phase of A1

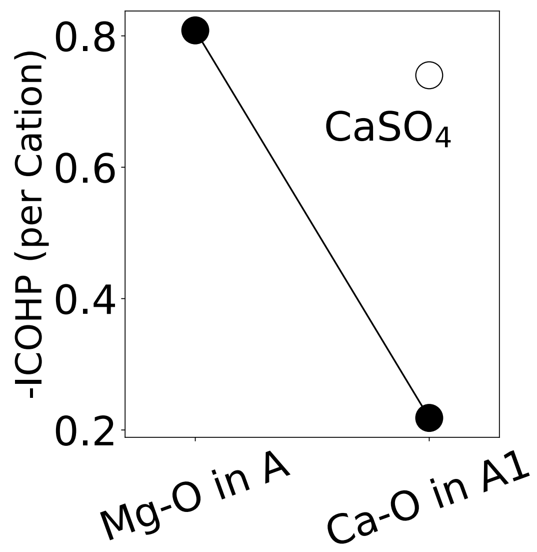

Figure S9. The calculated -ICOHP for different M-O bonds in NASICONs composition A and A1. As A1 is predicted to be unlikely synthesizable by DFT (E_{hull} = 48.7 meV/atom), the calculated -ICOHP per cation of the M-O bonds in competing phase is also plotted.

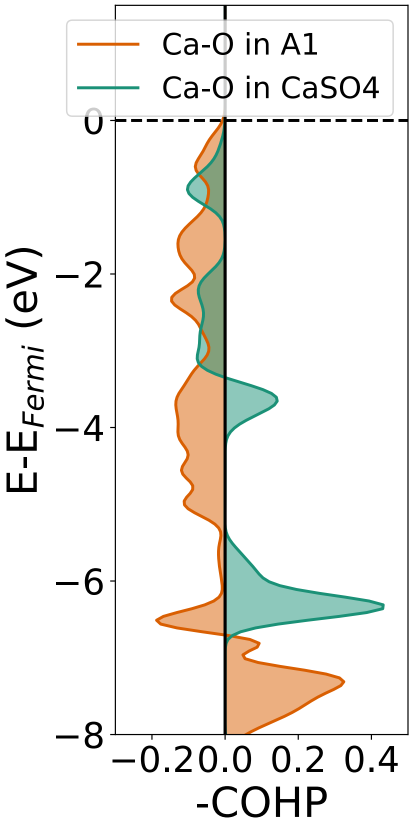

Figure S10. The calculated -COHP comparisons (normalized by M-O bond number) for Ca-O in A1 and $CaSO_4$.

References

1 Guin, M. & Tietz, F. Survey of the transport properties of sodium superionic conductor materials for use in sodium batteries. *Journal of power sources* **273**, 1056-1064 (2015).

2 Goodenough, J. B., Hong, H.-P. & Kafalas, J. Fast Na+-ion transport in skeleton structures. *Materials Research Bulletin* **11**, 203-220 (1976).

3 Losilla, E. R. *et al.* Understanding Na mobility in NASICON materials: a Rietveld, 23Na and 31P MAS NMR, and impedance study. *Chemistry of materials* **10**, 665-673 (1998).

4 Ma, Q. *et al.* Scandium-substituted Na3Zr2 (SiO4) 2 (PO4) prepared by a solution-assisted solid-state reaction method as sodium-ion conductors. *Chemistry of materials* **28**, 4821-4828 (2016).

5 Zhang, Z. *et al.* A Self‐Forming Composite Electrolyte for Solid‐State Sodium Battery with Ultralong Cycle Life. *Advanced Energy Materials* **7**, 1601196 (2017).

6 Cherkaoui, F., Villeneuve, G., Delmas, C. & Hagenmuller, P. Sodium motion in the Nasicon related Na1+ xZr2− xInx (PO4) 3 solid solution: An NMR study. *Journal of Solid State Chemistry* **65**, 293-300 (1986).

7 Nicholas, V., Johnson, P. & Kingon, A. Conductivity measurements in the Nasicon system. *Solid state ionics* **17**, 351-357 (1985).

8 Zahir, M., Olazcuaga, R. & Hagenmuller, P. Crystal chemistry and ionic conductivity in Nasicon-type phases Na1+ xZr2− xYbx (AsO4) 3 with 0⩽ x⩽ 0.45. *Materials Letters* **2**, 234-236 (1984).

9 Wang, W., Wang, S., Rao, L., Lu, Z. & Yi, X. Study of Na1+x+yZr2−yNdySixP3−xO12 fast ion conductors. *Solid State Ionics* **28-30**, 424-426, doi:https://doi.org/10.1016/S0167-2738(88)80076-6 (1988).

10 Susman, S., Delbecq, C., Brun, T. & Prince, E. Fast ion transport in the NASICON analog Na3Sc2 (PO4) 3: Structure and conductivity. *Solid State Ionics* **9**, 839-844 (1983).

11 Feltz, A. & Barth, S. Preparation and conductivity behaviour of Na3MIIZr (PO4) 3,(MII: Mn, Mg, Zn). *Solid State Ionics* **9**, 817-821 (1983).

12 Cava, R., Vogel, E. M. & Johnson Jr, D. W. Effect of homovalent framework cation substitutions on the sodium ion conductivity in Na3Zr2Si2PO12. *Journal of the American Ceramic Society* **65**, c157-c159 (1982).

13 Delmas, C. *et al.* Ioni conductivity in Nasicon-type phases Na1+ xZr2− xLx (PO4) 3 (L= Cr, In, Yb). *Solid State Ionics* **3**, 209-214 (1981).

14 Fujitsu, S., Nagai, M., Kanazawa, T. & Yasui, I. Conduction paths in sintered ionic conductive material Na1+ xYxZr2− x (PO4) 3. *Materials Research Bulletin* **16**, 1299-1309 (1981).

15 Takahashi, T., Kuwabara, K. & Shibata, M. Solid-state ionics-conductivities of Na+ ion conductors based on NASICON. *Solid State Ionics* **1**, 163-175 (1980).

16 Squattrito, P. J. *et al.* Sodium and oxygen disorder in a scandium-substituted nasicon: A time of flight neutron powder diffraction study of Na2. 5Zr1. 8Sc0. 2Si1. 3P1. 7O12. *Solid state ionics* **31**, 31-40 (1988).

17 Saito, Y., Ado, K., Asai, T., Kageyama, H. & Nakamura, O. Ionic conductivity of NASICON-type conductors Na1. 5M0. 5Zr1. 5 (PO4) 3 (M: Al3+, Ga3+, Cr3+, Sc3+, Fe3+, In3+, Yb3+, Y3+). *Solid State Ionics* **58**, 327-331 (1992).

18 Wang, W., Li, D. & Zhao, J. Solid phase synthesis and characterization of Na3Zr2− yNb0. 8ySi2PO12 system. *Solid state ionics* **51**, 97-100 (1992).
19 Yue, Y., Zhou, F. & Pang, W. Hydrothermal crystallization and structural investigation of Na1+ 2xZr2− xMgx (PO4) 3 systems (0< x< 1). *Materials Chemistry and Physics* **35**, 28-30 (1993).
20 Yue, Y. & Pang, W. Hydrothermal synthesis and characterization of NaSn2(PO4)3. *Journal of Materials Science Letters* **11**, 148-149, doi:10.1007/BF00724673 (1992).
21 Wang, W. & Liu, X. Study on sodium fast ion conductors in the Na1+ 3xAlxHf2− xSi2xP3− 2xO12 system. *Solid state ionics* **89**, 165-168 (1996).
22 Veríssimo, C. *et al.* Ionic conductivity and structural characterization of Na1. 5Nb0. 3Zr1. 5 (PO4) 3 with NASICON-type structure. *Solid State Ionics* **100**, 127-134 (1997).
23 Fuentes, R., Figueiredo, F., Marques, F. & Franco, J. Influence of microstructure on the electrical properties of NASICON materials. *Solid State Ionics* **140**, 173-179 (2001).
24 Maldonado-Manso, P., Aranda, M. A., Bruque, S., Sanz, J. & Losilla, E. R. Nominal vs. actual stoichiometries in Al-doped NASICONs: A study of the Na1. 4Al0. 4M1. 6 (PO4) 3 (M= Ge, Sn, Ti, Hf, Zr) family. *Solid State Ionics* **176**, 1613-1625 (2005).
25 Zhang, Q., Wen, Z., Liu, Y., Song, S. & Wu, X. Na+ ion conductors of glass–ceramics in the system Na1+ xAlxGe2− xP3O12 (0.3⩽ x⩽ 1.0). *Journal of alloys and compounds* **479**, 494-499 (2009).
26 Song, S., Duong, H. M., Korsunsky, A. M., Hu, N. & Lu, L. A Na+ superionic conductor for room-temperature sodium batteries. *Scientific reports* **6**, 1-10 (2016).
27 Lu, Y. *et al.* A high‐performance monolithic solid‐state sodium battery with Ca2+ doped Na3Zr2Si2PO12 electrolyte. *Advanced Energy Materials* **9**, 1901205 (2019).
28 Jolley, A. G., Cohn, G., Hitz, G. T. & Wachsman, E. D. Improving the ionic conductivity of NASICON through aliovalent cation substitution of Na 3 Zr 2 Si 2 PO 12. *Ionics* **21**, 3031-3038 (2015).
29 Samiee, M. *et al.* Divalent-doped Na3Zr2Si2PO12 natrium superionic conductor: Improving the ionic conductivity via simultaneously optimizing the phase and chemistry of the primary and secondary phases. *Journal of Power Sources* **347**, 229-237 (2017).
30 Yahia, H. B. *et al.* Sodium intercalation in the phosphosulfate cathode NaFe2 (PO4)(SO4) 2. *Journal of Power Sources* **382**, 144-151 (2018).
31 Vogel, E., Cava, R. & Rietman, E. Na+ ion conductivity and crystallographic cell characterization in the Hf-nasicon system Na1+ xHf2SixP3− xO12. *Solid State Ionics* **14**, 1-6 (1984).
32 Deng, Y. *et al.* Crystal structures, local atomic environments, and ion diffusion mechanisms of scandium-substituted sodium superionic conductor (NASICON) solid electrolytes. *Chemistry of Materials* **30**, 2618-2630 (2018).
33 Zhu, Y., Li, L., Li, C., Zhou, L. & Wu, Y. Na1+ xAlxGe2− xP3O12 (x= 0.5) glass–ceramic as a solid ionic conductor for sodium ion. *Solid State Ionics* **289**, 113-117 (2016).
34 Guin, M., Tietz, F. & Guillon, O. New promising NASICON material as solid electrolyte for sodium-ion batteries: Correlation between composition, crystal structure and ionic conductivity of Na3+ xSc2SixP3− xO12. *Solid State Ionics* **293**, 18-26 (2016).

35 Khakpour, Z. Influence of M: Ce4+, Gd3+ and Yb3+ substituted Na3+ xZr2-xMxSi2PO12 solid NASICON electrolytes on sintering, microstructure and conductivity. *Electrochimica Acta* **196**, 337-347 (2016).
36 Naqash, S., Tietz, F. & Guillon, O. Synthesis and characterization of equimolar Al/Y-substituted NASICON solid solution Na1+ 2x+ yAlxYxZr2− 2xSiyP3− yO12. *Solid state ionics* **319**, 13-21 (2018).
37 Devroye, L. & Lugosi, G. *Combinatorial methods in density estimation*. (Springer Science & Business Media, 2012).
38 Massart, P. The tight constant in the Dvoretzky-Kiefer-Wolfowitz inequality. *The annals of Probability*, 1269-1283 (1990).
39 Devroye, L., Györfi, L. & Lugosi, G. *A probabilistic theory of pattern recognition*. Vol. 31 (Springer Science & Business Media, 2013).
